# Simple and Complex Working Memory Tasks Allow Similar Benefits of Information Compression

**DOI:** 10.5334/joc.31

**Published:** 2018-05-25

**Authors:** Fabien Mathy, Mustapha Chekaf, Nelson Cowan

**Affiliations:** 1 Université Côte d’Azur, CNRS, BCL, UMR 7320, FR; 2 University of Missouri-Columbia, US

**Keywords:** Working memory, Short-term memory, Memory

## Abstract

Complex working memory span tasks were designed to engage multiple aspects of working memory and impose interleaved processing demands that limit the use of mnemonic strategies, such as chunking. Consequently, the average span is usually lower (4 ± 1 items) than in simple span tasks (7 ± 2 items). One possible reason for the higher span of simple span tasks is that participants can take advantage of the spare time to chunk multiple items together to form fewer independent units, approximating 4 ± 1 chunks. It follows that the respective spans of these two types of tasks could be equal (at around 4 ± 1) if stimulus lists exclusively used nonchunkable stimulus items. To manipulate the chunkability of the stimulus lists, our method involved a measure of their compressibility, i.e., the extent to which a pattern exists that can be detected and used as a basis of chunk formation. We predicted an interaction between the types of tasks and chunkability/compressibility, supporting a single higher span for the condition in which a simple span task was combined with chunkable items. The three other conditions were predicted to prevent chunking processes, either because the interleaved processing task did not allow any chunking process to occur or because the noncompressible material inherently limited the chunkability of information. The prediction that chunking is important solely in simple spans was not confirmed: Effects of information compression contributed to performance levels to a similar extent in both tasks according to a theoretically-based metric. This result suggests that *i*) complex span tasks might overestimate storage capacity in general, and *ii*) the difference between simple and complex span performance levels must rest in some mechanism other than prevention of a chunking strategy by the interleaved processing task in complex span tasks.

Working memory is information temporarily held in mind, which can be useful to complete a diverse range of cognitive tasks (Baddeley & Hitch, 1974). Working memory (WM) capacity is often assessed with complex working memory span tasks (complex span tasks for short; e.g., reading span, operation span; see Conway et al., 2005). Complex span tasks interleave a memory task (requiring one to remember a set of items in the correct order) with a secondary processing task (e.g., judge the correctness of equations). The dual nature of complex span tasks is central to Baddeley’s (2000) model, which distinguishes a storage component and a central attentional control system, but also to more recent models (Barrouillet, Bernardin, & Camos, 2004; Engle, 2002). One advantage for conducting complex span tasks is that they are predictive of intelligence (Conway, Cowan, Bunting, Therriault, & Minkoff, 2002; Unsworth & Engle, 2007), which notably requires both memorizing and manipulating several pieces of information to solve a problem.

Because complex span tasks were designed to include an interleaved processing task, the average span is usually lower than the average 7 ± 2 items observed in simple short-term memory span tasks (simple span tasks for short), that is, around 4 ± 1 items (Cowan, 2001). As a result, research has suggested that simple span tasks and complex span tasks measure different processes (Conway et al., 2002; Unsworth & Engle, 2006). Put simply, simple span tasks are commonly considered typical tasks for measuring short-term memory (which is considered passive and dedicated to item memorization), while complex span tasks are usually considered typical measures of working memory (which is assumed to involve information processing beyond that needed for storage). To understand how information processing differs in simple versus complex span, it would help to understand why these types of task yield such different spans. Our goal was to pursue one plausible hypothesis, that the diversion of processing away from storage in complex span reduces the extent to which information to be remembered can be chunked in that kind of task.

Chunking is a cognitive process that can be characterized by the formation of a unit from different pieces of information. It can be underpinned by various mechanisms such as deliberate long-term memory retrieval or automatic perceptual grouping (Gobet et al., 2001). One potential mechanism (among others) is that chunks can be formed by compressing information to reduce several pieces of information into a more minimal expression. Compressibility of information is the extent to which existing patterns can be detected (Chater & Vitányi, 2003) and used as a basis of chunk formation. This mechanism of information compression allowing more items to be stored in memory has been shown to occur rapidly in simple span tasks in an on-line manner (Chekaf, Cowan, & Mathy, 2016), and can benefit capacity in visual working memory experiments in the long term (Brady, Konkle, & Alvarez, 2009). Although chunking has long been known to be important to account for the 7 ± 2 upper-bound estimate of short-term memory (Miller, 1956), we wondered whether this on-line compression might be impossible in a complex span task. Compressibility was used in the present study to predict on-line chunking, assuming that chunking can make use of a compression process to form new units.^1^

The compression account of chunking processes in simple span tasks is one in which participants can detect regularities in the stimulus set, which are used to pack a few stimuli together in fewer independent units (Chekaf, Gauvrit, Guida, & Mathy, in press; Mathy & Feldman, 2012). One example of regularity in a digit series would be the series of two digits ‘19’, which could point to a single idea (e.g., the *19^th^* century). Two random letters can also coincidently present a regularity if they evoke a word, as well as several visual stimuli can make regular patterns. Forming three or four groups is in fact sufficient to artificially increase capacity to 7 items (e.g., 1984527 = ‘1984’-5-2-7), but there may be a greater chance that this process occurs when there is no interleaved processing task assumed to disrupt chunking. The hypothesis investigated in the current study is that, if information compression or chunking in simple span is the reason why it exceeds the performance levels observed in complex span, then we should observe a similar capacity (i.e., 4 ± 1 items) for simple and complex span tasks if stimulus recoding is hindered in both cases.

To manipulate the compression process in our task, we derived our task from the categorization domain, which has shown that information can be re-encoded when compressible (Feldman, 2000). We used basic visual stimuli (colored shapes), such as 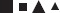, which contained regularities or not depending on experimental conditions. For instance, this series is regular in that the color dimension can be used to recode the objects and because the shapes can be grouped by pairs (and with a possibility to order sizes within shapes). Regularity in this frame can be viewed as any kind of redundant information that can be potentially recoded to minimize information. For instance, instead of three features per shape (color, shape, size), which totalizes 12 pieces of information, the smaller expression “black (squares-triangles, large-small within shapes)” is an example of algorithmic compression which benefits from the available structure.

Our experiment directly compared a simple span task to a complex span task, using chunkable *vs*. nonchunkable sequences of stimuli. Chunkability was estimated by the compressibility of our stimulus lists. We expected to find that the compressibility of the materials would make a difference only for simple span tasks, under the assumption that compression of information is not possible in complex span tasks (because of the secondary processing task, which is used to switch the focus of attention away from the primary task). This prediction assumes that impaired processing activities allocated to the memory items in complex span tasks (Barrouillet, Portrat, & Camos, 2011) implies impaired chunking processes. The predicted interaction was therefore thought to support only a higher span for the simple span task in the chunkable condition, in which compressible information could be processed. The three other conditions were predicted to prevent chunking processes, either because the interleaved processing task did not allow any chunking process to occur or because the noncompressible material inherently limited the chunkability of information.

## Method

**Figure 1:**
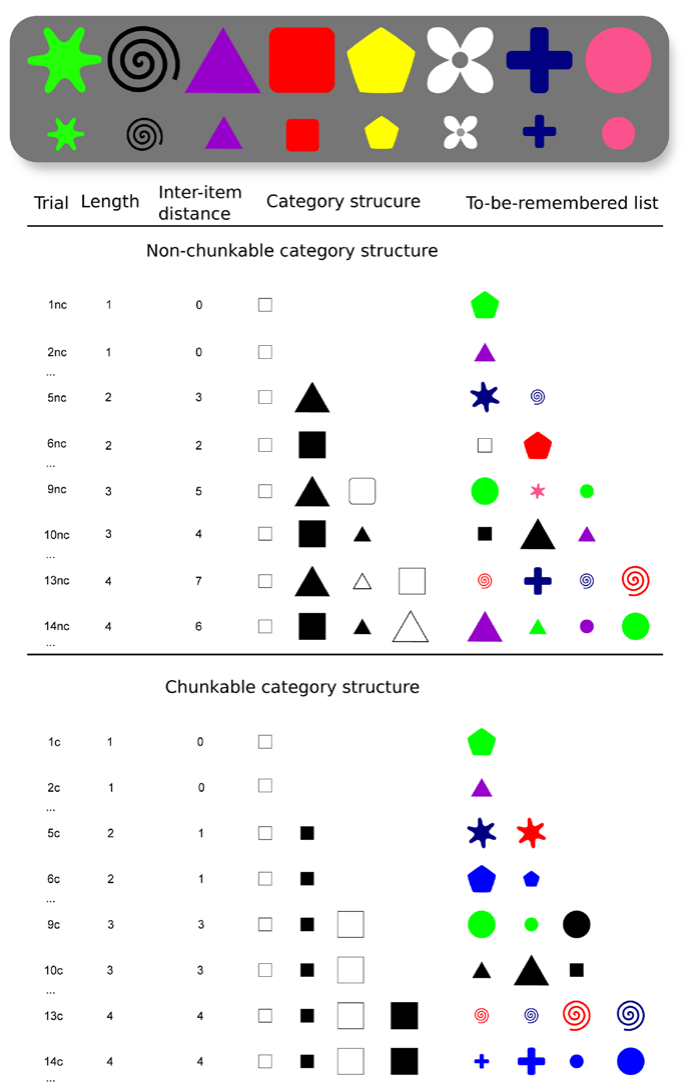
(Top) A sample of stimuli based on eight shapes, eight colors and two types of sizes. (Bottom) Table showing a sample of trials. Trials are indexed using ‘nc’ and ‘c’ to easily refer to the respective non-chunkable and chunkable condition in the text. The first half of the table shows the sequences of the non-chunkable condition. The second half of the table shows the sequences of the chunkable condition. For each sequence length, we chose to represent only two trials. Dimension values were chosen randomly for each trial. For instance, the given category structure 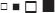 (i.e., square, small first, white-black within sizes) could be converted into either 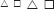 (i.e., white, small first, triangle-square within sizes), or 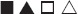 (i.e., large, black first, square-triangle within colors), etc. The preceding example only involve the dimension values square, triangle white and black, but again, the dimension values were actually randomly picked among the 8 shapes and the 8 colors shown in the top panel, and using two sizes randomly.

To manipulate the compression process in our span task, we limited the relational information in a set of basic visual stimuli (such as colored shapes), using simple colors and simple shapes. More specifically, we used lists of visual, categorizable, artificial three-dimensional stimuli, with two-valued/Boolean discrete features for the shapes, sizes, and colors shown in Figure 1. For a given three-dimensional set, we selected sets with stimuli of the lowest relational information, a manipulation that was hypothesized to prevent chunking. Let us illustrate with the three-dimensional set of objects: 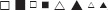. A compressible subset of four objects would be: 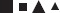, because the color dimension is sufficiently diagnostic to discriminate black objects from white objects. A stimulus list based on this subset offers the possibility of re-encoding the sequence using the simple rule ‘black’. The sequence 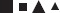, in which order matters, can be described by a simple rule using the ‘black’ feature (to recode the entire subset, regardless of order) and the ‘square-triangle’ order which can be combined with a ‘large-first’ description within each shape. By contrast, a less compressible subset of objects would be: 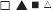. The heterogeneity of these four objects that makes the category structure complex can be measured by the difficulty of compressing information, accounting for the difficulty of recoding the stimuli into a more compact representation (Feldman, 2000). In other words, there is no simple, hierarchical rule that explains the sequence of shapes/colors of this subset. More homogeneous category sets produce a lower information load and as such, they are more compressible and can be easily re-encoded (or “chunked”) to facilitate recall (Chekaf et al., 2016). To sumarize, Figure 1 (bottom) shows two differences between chunkable and non-chunkable. (1) The chunkable lists can be described with fewer total number of features, AND (2) the chunkable lists are arranged in a serial order that allows the compressibility to be easily discovered (Mathy & Feldman, 2009).

Hereafter, we call the simple compressible homogeneous sequences “chunkable” and the complex sequences “non-chunkable” (or “less-chunkable” when more convenient). The reason we do this is that we assume that (1) capacity is roughly 3 or 4 chunks and (2) increases in performance for more compressible lists does not result from a change in chunk capacity (see Cowan, Rouder, Blume, & Saults, 2012) but from an effective increase in the size of chunks. Even if performance sometimes results from graded associations between items rather than discrete chunks, the chunk vocabulary conveniently expresses the amount of increase in performance with more compressible lists. Accordingly, four conditions were constructed: a simple span task using chunkable material, a complex span task using chunkable material, a simple span task using non-chunkable material, and a complex span task using non-chunkable material.

We predicted that the simple span task could only have a beneficial effect on recall when some of the information could be re-encoded, while such a benefit could not occur when no information (or little information) could be re-encoded. Conversely, a complex span task offers no opportunity to recode the regular patterns in the chunkable condition because attention is directed away during the interleaved processing task. Therefore, we predicted an interaction between task and compressibility, supporting only a higher span for the simple span task in the chunkable condition. To test the size of the interaction, we planned on running a Bayesian analysis to compare the quantity of material chunked in the four conditions, and particularly using a chunking score reflecting the quantity of materials chunked in the simple span task and the complex span task. A strong interaction should be supported by a smaller chunking score for the complex task.

*Participants*. Ninety-four students (*M* = 23 years old, *sd* = 5.3) enrolled at the Université Côte d’Azur volunteered to participate in the experiment. Estimate of sample size was computed based on the difference observed in our previous study for proportion correct between the most chunkable condition and the least chunkable condition. We obtained 75 < *N* < 105, depending on *η* varying between .40 and .55, with .55 being the value obtained in our previous study, for a power of .80.

*Stimuli*. Our stimuli varied according to three two-valued/Boolean dimensions (shape, size and color, the three dimensions typically used by category learning researchers to build canonical stimulus sets; Love & Markman, 2003). We used only two values per dimension within each trial (Figure 1, bottom). For each trial, a random combination of two shapes (among eight different ones), two colors (among eight different ones), and two sizes made a set of eight possible objects. We restricted the size dimension to two different values (large *vs*. small, i.e., 280 × 280 pixels *vs*. 140 × 140 pixels) across lists because participants had trouble identifying intermediate values during our pre-tests. The use of eight shapes, eight colors, and two sizes was sufficient to generate 1568 possible sets of eight objects, which limited proactive interference between trials (a sampled combination of features is given in Figure 1, top).

The participant did not know in advance which of the dimensions would be the most relevant to the categorization process. Dimension values were chosen randomly for each of the lists presented, so as to vary the possible combinations of dimensions (shapes, sizes, and colors) across lists, while preserving the same category structure (shown in Figure 1). The probability that a participant would come across two identical sets of features between two lists during the experiment was assumed to be very low.

*Procedure*. The experiment was a 2 × 2 within-subject design. Each participant attempted all four blocks (chunkable simple span task, non-chunkable simple span task, chunkable complex span task, non-chunkable complex span tasks), the order of which was counterbalanced across participants (i.e., 24 possible orders; 96 participants were needed to perfectly balance the design). Each block comprised several lists of stimuli and recall occurred after each list. The participants were informed that they were required to memorize, in correct order, each list of stimuli. A list of stimuli (e.g., a small blue square and a large blue square) was chosen from a random combination of two shapes (e.g., all the stimuli resulting from the combination of small *vs*. large, blue *vs*. red, and square *vs*. circle objects). The stimuli in a given sequence were displayed serially in the center of the screen for one second each (e.g., for a two-stimulus list, a small blue square followed by a large blue square). Difficulty of each sequence was estimated following the compressibility metric described by Chekaf et al. (2016) and based on Feldman (2000). This metric simply makes use of disjunctive normal formulas (a disjunctive list of conjunction of features) to compute the minimal number of features that reduce the uncompressed lists of objects (which list verbatim all of the features of the constituent objects within lists).

After the list of items was presented, the response screen showed the whole set of eight objects from which the subset had been selected. The response screen showed in randomly-determined positions eight response choices: the *k* to-be-recalled stimuli and the 8 – *k* remaining distractor objects. Participants were required to recall the list of items and to reconstruct their order. The participant made selections by clicking on the objects to recall the items in the correct order. This recall procedure is similar to that of the visual short-term-memory serial report task (Avons & Mason, 1999; Smyth, Hay, Hitch, & Horton, 2005). The stimuli were underlined using a white bar when the user clicked on them. There was no timing constraint for recall. The participant could move on to the next sequence by pressing on the space bar.

The 8 – *k* remaining distractor objects in the test screen allowed us to compute the compressibility properly. For instance, for Trial #14nc shown in Figure 1, the recall screen included a large green triangle, a small purple triangle, a small green circle, and a large purple circle as the new items, in addition to the four stimuli (large purple triangle, small green triangle, a small purple circle, and a large green circle). Trial #14c shown in Figure 1 included the four red objects in addition to the four blue stimuli. The compressibility of the memoranda was therefore intentionally correlated with retrieval demands of the trials. Following the previous example, the new items of trial #14nc are logically more interferent with the memoranda because the features of the lures overlap with those of the to-be-recalled stimuli. Conversely, the red lures could be less confounded with the blue stimulus objects in #14c. Because ‘blue’ is a simple description of the memoranda, the opposite category is necessarily also simple (i.e., ‘red’). The fact that every description and its complement have the same complexity is generally referred to as parity.

The lists were displayed using ascending presentation of length (length varied progressively from 1 to 8 items), as in the digit spans used in neuropsychological tests. Trial length 1 was only used as a warmup. For instance, our experiment used the same number of repetitions per length as the digit span of the WISC or WAIS. A block automatically stopped after four errors within a given list length (an error was simply the incapacity of the participant to recall back the sequence entirely in perfect order). Participants were given four trials per length *L*. They were also informed that the first three trials in each block would be treated as practice trials and then discarded from the analysis. After this warmup, there was four trials per list length in each condition.

When the task was a simple span task, there was a 500ms inter-item interval. When the task was a complex span task, we used the operation span (OS) task procedure. In OS, participants are required to perform mathematical operations between memory items (see Conway et al., 2005; Kane et al., 2004). An equation was displayed on the screen (e.g., “7 + 2 = 10”) before each to-be-remembered item was presented (equations were read quietly). The participant had three seconds to judge the equation by clicking a button (true or false), before the next item was displayed. The equation disappeared after the participant made a response, just before the next item was displayed. This interleaved processing task was thought to prevent participants from chunking freely.

For the non-chunkable simple span, for a given list length, the most incompressible lists alternated with less incompressible lists; otherwise, chunks would have exhibited too much similarity across the experiment. For instance, in Figure 1, Trial #10nc shows the most incompressible three-object set, with a first 2-feature difference (size and color, between the little white square and the large black square) followed by a second 2-feature difference (size and shape, between the large black square and the small black triangle), whereas Trial #9nc shows a less incompressible 3-object set, ordered using a 3-feature difference followed by a 2-feature difference to make the chunking process harder. The inter-item distance (the summed number of feature differences between objects) is convenient to describe the relationships between features, but Feldman (2000, 2003) describes more precisely how the features can be redescribed to compress the sum of information in each set of objects (the compression process is not always related to inter-item distance). For instance, “small white square, small black square” can be reduced to two features (“small square”), whereas “small white square, large black triangle” cannot be reduced to less than six features. Here, for instance, the overall description of the three objects in Trial #9nc requires a minimal logical expression of 5 features, instead of 8 features for #10nc; see (Feldman, 2003). This measure of compressibility only serves here to predict the chunkability of a category set (exact order such as ‘first white’ still requires one more piece of information in the experimental context). Overall, all of the category structures of a given length were chosen to be less compressible in the non-chunkable condition than in the chunkable condition.

*Scoring*. To compute an estimate of the span in each condition, a value of .25 was scored for each perfectly correct serial report of all the memory items within a trial.^2^ For instance, a participant recalling only 3 out of 4 sequences of one object would be granted a span of .75, if failing totally for longer sequences. When a subject obtained 4, 4, and 3 trials correct at lengths 1, 2 and 3 respectively, then the span was equal to (4 + 4 + 3)/4 = 2.75. When a subject obtained 4, 3, and 2 trials correct at lengths 1, 2 and 3 respectively, then the span was equal to (4 + 3 + 2)/4 = 2.25.

## Results

*Concurrent task*. After averaging by participant, a paired-samples *t*-test on accuracy at the concurrent task (i.e., on the operations to be judged in the complex span task) showed no difference between the chunkable (87%, *sd* = .08) and the nonchunkable (86%, *sd* = .07) conditions, *t*(93) = 1.49, *p* = .14. No significant linear trend (using repeated-measures ANOVA with a polynomial linear trend, or simply using correlations) was found between length of the memoranda and accuracy for both the chunkable and nonchunkable conditions (again, after averaging by participant).

**Figure 2:**
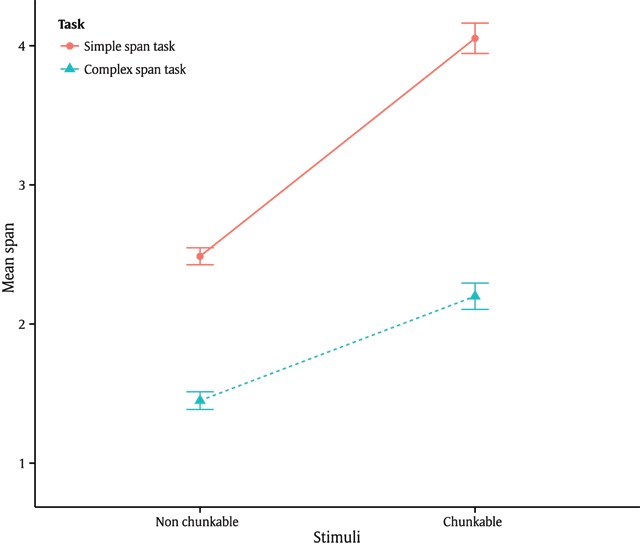
Mean span by procedure (simple vs. complex span tasks) and category set complexity (chunkable vs. non-chunkable). Error bars represent +/– one standard error.

*Effect of task procedure and category-set complexity*. An ANOVA was performed with task procedure (simple *vs*. complex span task) and category-set complexity (chunkable *vs*. non-chunkable) as repeated factors, using the mean span as the dependent variable. In the simple-span and complex-span conditions, the mean spans were 3.45 (*sd* = .77) and 1.94 (*sd* = .70), respectively. This analysis yielded a significant effect of task procedure (*F*(1,93) = 421.85, *p* < .001, *η*^2^*_p_* = .82). The mean spans when the lists of objects were chunkable and non-chunkable were 3.35 (*sd* = .83) and 2.08 (*sd* = .49), respectively. Analysis showed a significant effect of category-set complexity (*F*(1,93) = 268.22, *p* < .001, *η*^2^*_p_* = .74). We also found a significant interaction between the two factors (*F*(1,93) = 40.29, *p* < .001, *η*^2^*_p_* = .30). The spans in all four conditions are shown in Table 1 and Figure 2. The four simple effects were all found significant (*t*(93) > 2.97, *p* < .01), so the interaction was due to the greater difference between Nonchunkable and Chunkable in the simple span task condition.

**Table 1:** Mean span (and standard errors), by procedure (simple vs. complex span tasks) and category set complexity (chunkable vs. non-chunkable), and mean chunking scores.

	Non-Chunkable	Chunkable	Chunking score (global)	Chunking score (individual)

Simple Span	2.49(.06)	4.05(.11)	1.63	1.70(.05)
Complex Span	1.45(.06)	2.2(.09)	1.52	1.76(.12)

*Note.* The global chunking scores are simply based on the two average values of the same line in the table (e.g., 4.05/2.49 = 1.63). The individual chunking scores were figured out on a ratio separately for each participant (standard errors are in parentheses).

This interaction seems to suggest that the chunking benefit was greater for simple vs. complex span tasks since effects of the two factors were not additive. However, a better way to test the benefit of information compressibility is to calculate how much information could be packed in diverse conditions compared to a baseline. Here, if the increased span due to chunking was 2.2/1.45 =1.52 (Table 1), while the increased span due to the simple span task was 2.49/1.45 = 1.72, the relative increase due to both factors should be 1.52 × 1.72 = 2.6. The expected number 2.6 represents how much more information should be packed when the two factors are combined. Given a baseline capacity of 1.45 in the less favorable condition for processing information (Table 1), we would expect a span equal to 1.45 × 2.6 = 3.8 when both effects occur. Since the observed increase was actually 4.05/1.45 = 2.79, the observed interaction is very close to the expected multiplicative effect of 2.6. The next analysis therefore tested whether more information was compressed in the chunkable stimuli of the simple span task condition, which would be the case if the span in this condition significantly exceeded a simple multiplicative effect.

*Multiplicative chunking effect test*. To test whether compressibility had roughly the same effect in the simple and complex-span tasks, we measured the average chunking performance for the entire task as the following ratio: the average span of the chunkable condition divided by the average span of the non-chunkable condition, for each type of task. The span here still refers to the one computed using the method described above in subsection Scoring. We obtained the following average chunking performance (the four following numbers are the average span values for the four conditions obtained across participants): Simple span: 4.05 (chunkable)/2.49 (non-chunkable) = 1.63; complex span: 2.2 (chunkable)/1.45 (non-chunkable) = 1.52. Compressibility in the lists of objects had therefore a multiplicative effect on recall, insofar as participants recalled about 1.5 times more items when there were regularities than when there were not.

Individual chunking performance were then calculated as follows for every participant: the span of the chunkable condition divided by the span of the non-chunkable condition, for each type of task. These individual chunking scores were submitted to a Bayesian analysis. In this case, mean participant’s chunking scores were even closer than using the average values for the four conditions, that is 1.70 (*sd* = .51) for the Simple span task and 1.76 (*sd* = 1.19) for the Complex span task. Using JASP,^3^ we tested the null hypothesis that the chunking scores of the simple and complex tasks would be the same. The appropriate alternative hypothesis was that the chunking score for complex span tasks is lower than the chunking score for simple span tasks, i.e., that stimuli in simple span tasks are more highly chunkable. The Cauchy Prior with a width of .707 was used to test the alternative hypothesis (the program’s default). With that alternative hypothesis (using just half of the Cauchy Prior), the Bayes factor was 12.69 in favor of the null hypothesis over the alternative hypothesis. A less theoretically-guided test would be a two-tailed test, the alternative now being that the chunking scores could differ in either direction. With that alternative hypothesis, the data came out with a Bayes factor of 7.58 in favor of the null hypothesis. Both exceed the ratio of three that seems to be a standard convention for a sufficiently decisive finding. This other surprising result leads us to believe that chunking in immediate memory is a deep process that is resistant to interleaved processing tasks, at least deeper than what our initial hypotheses suggested.

To ensure that other effects that we report below produce a Bayesian ratio of 3.0 or higher in favor of the alternative hypotheses, we also used JASP with default priors to run a Bayesian repeated measures ANOVA with task procedure (simple *vs*. complex span task) and category-set complexity (chunkable *vs*. non-chunkable) as repeated factors. We obtained a Bayes factor of 1.21*e*^79^ in favor of the alternative model (a model including a main effect of task procedure, a main effect of category-set complexity, and an interaction between the two), against the null model. Also, the Bayes factor for this alternative model against a model without the interaction component was 1.22*e*^6^. Other simpler models (one model including only an effect of task procedure, another model including only an effect of category-set complexity, or else another model including both effects without interaction) were all much better than the null model, but there was more evidence for the full model. Analysis based on model averaging (combining models that included a particular effect, either task procedure, category-set complexity, or their interaction) showed that changes from prior to posterior inclusion odds were all decisive (Bayes factor > 10*e*^2^, meaning that the Bayesian analysis accounted better for our results by including all of the factors in play, once the candidate models were updated with our data).

## Discussion

We found no fundamental difference between the simple and complex span tasks regarding the ability to recode information. We showed that a chunking process can operate in complex span tasks in a manner comparable to simple span task inasmuch as we found a ratio of items retained of about 1.7 in the compressible:incompressible conditions in both simple and complex span procedures. Compression seems more ubiquitous than predicted, meaning the formation of chunks can presumably occur even in complex span tasks. This could mean that interleaved processing tasks simply reduce the time to process the memory items and that chunking and related recoding processes can still sufficiently occur even in complex span tasks (and therefore may be more automatic than expected). In other words, the more limited time to process the memory items during a complex span task (than during a simple span task) could simply impair chunking proportionally.

The idea that chunking can occur in complex span tasks is plausible. For instance, Portrat, Guida, Phénix, and Lemaire (2016) showed that chunks could be formed in complex span tasks, but their material used chunks already formed in long term memory (such as the acronym PDF). The authors showed that, although not presented in immediate succession because of the concurrent task, the different constitutive elements of a stimulus list of letters could be recognized as chunks. Because several items need to be reactivated for potential chunking, the authors concluded that complex attentional processes must be at play in working memory. Our finding completes the results of this previous study by showing that *new* chunks can be formed within the attentional constraints imposed by complex span tasks. Our observation that there is no fundamental difference between the simple and complex span tasks is also consistent with previous findings which have used a correlational approach to compare different estimates of the span (Colom, Rebollo, Abad, & Shih, 2006; Colom, Shih, Flores-Mendoza, & Quiroga, 2006; Martínez et al., 2011; Unsworth & Engle, 2007).

Secondly, we showed that the average span was less than three objects using a simple span task with incompressible sequences. Although it may seem troubling to obtain such an unexpectedly low span, such a concern would be based on the assumption that the limit should apply to a certain fixed number of objects, with each object including all of its features bound together (Luck & Vogel, 1997). However, there is more recent evidence that features must be held in mind separately (Hardman & Cowan, 2015; Oberauer & Eichenberger, 2013). It might be possible that participants retained about four objects, but some objects might not be perfectly encoded.

One possibility is that there was too much regularity in our Chunkable condition (because of the parity between the to-be-recalled objects and the lures) and participants may have used a rule such as “stimuli in general do not vary in one of three dimensions” across the chunkable conditions as a strategy to retain only two dimensions among three. One could argue that such a rule could have simplified the recall process with limited chunking during encoding for a particular trial since the rule could be used across trials. If true, we believe that it still does not undermine the idea that chunkability was manipulated in our experiment. Although such a rule can help determine information to encode a chunkable set of items (this is actually the core of our hypothesis that compressible structures represent less information than the original sets of individual items), one important aspect of our method is that the task required to reconstruct order. This reconstruction process required to encode information about the entire sequence of items. For instance, if two blue crosses were presented, followed by two blue circles (with the order ‘small first, large second’ within shapes), information about the exact position of all objects was necessary to encode the optimal chunk ‘crosses first, small first within shapes’. Therefore, it was not sufficient for participants to expect one diagnostic feature (before the trial began) and to notice for this particular trial that all items were eventually ‘not white’ to perform perfectly. But more importantly, if such a rule makes some of the potential answers ineligible, it does not simplify encoding until the participants is presented with the particular sequence of objects within which the specific order is unique. For instance, if a ‘small blue cross’ would be presented first, there were still four possible sequences (blue crosses, white crosses; blue crosses, blue circles; small blue, small white; small cross, small circle) associated with each potential pairs of shape and color features (we chose here white and circle, but these features were randomly drawn). Then, after the second object was chosen (for instance ‘small blue cross’), again, the third critical feature was not yet randomly drawn and could have been either circles or white objects (and again, these two features taken for the sake of the example could be chosen randomly among many). Also, a general rule could also be applied to nonchunkable series since there were never two consecutive objects related by more than one feature in common. If such rules were used, they could therefore be used no matter the condition, but their use would have implied rapid deductive logic which does not seem easier than just encoding objects as they come.

Still, we reanalyzed our data published in *Cognition* in 2016 to provide further evidence that our procedure is suitable. The reason is that the chunkable trials were randomly mixed with nonchunkable trials in this old set, which supposedly made the use of a general rule applied to a given condition unstrategic. We selected the exact same trials and we adopted a scoring procedure that fitted both data sets. The result shows strikingly similar performance (in the simple span task conditions, because the old experiment did not use a complex span task) between the two data sets: we observed a span of 2.43 (present data) vs 2.52 (old data) when nonchunkable, and a span of 3.86 (present data) vs 3.66 (old data) when chunkable (the two respective *t*-tests are not significant).

Finally, aside from potential alternative explanations for why our chunkable series were recalled more easily, our result more generally focuses on whether information can be manipulated in complex span tasks in the same extent as in simple span tasks. We observed that effects of information compression contributed to performance levels to a similar extent in simple and complex span tasks. Chunking processes could still operate in complex span tasks, whereas such tasks were designed to divide processing of the memoranda by imposing an additional processing task performed in-between each of the to-be-recalled items. If information can be manipulated in complex working memory span tasks, it simply means that attention might not be directed away from storage completely in such tasks and that undivided processing of the memoranda is not needed to form a new chunk. This result suggests that *i*) neither simple nor complex spans can be assumed to reflect working memory capacity in the absence of chunking *ii*) the difference between the two types of task must rest in some mechanism other than prevention of mnemonic strategies by the interleaved processing task in complex span. Our conclusion is that observation of the spans in non-chunkable vs. chunkable series rule out that chunking is important solely in simple span tasks, and thus recoding cannot be uniquely responsible for the large difference in the spans usually observed in simple and complex span tasks.

One potential limitation of the present study is that visual objects were used as memoranda. Even though visuo-spatial complex span tasks have been used in the literature (e.g., Unsworth & Engle, 2007), many other existing complex span tasks are verbal in nature (e.g., reading span, operation span, etc.). After citing research indicating evidence from visuospatial tasks that complex spans and simple spans are more correlated than for verbal tasks, Kane et al. (2004) showed that their four-factor model could clearly separate verbal and spatial complex spans and verbal and spatial simple spans (p. 203). In that respect, we think that visuo-spatial tasks can sufficiently help discriminate the two types of tasks. However, data from further experiments using verbal material would help extend our result.

In the future, it could also be important to study other presentation time lags. If the main reason for the difference observed between simple spans and complex spans is that participants take advantage of the greater amount of spare time in simple span tasks to encode information, experiments to be conducted with the highest priority would target accelerated presentations such as those used in the change-detection paradigm (Rouder, Morey, Morey, & Cowan, 2011). This would offer a larger time scale range (across all tested types of working memory tasks: change-detection, complex span, simple span) that would benefit not only theories of visual working memory (Luck & Vogel, 1997; Wheeler & Treisman, 2002), but also conceptions of binding, grouping (Farrell, 2012) and chunking that are not yet considered connected processes. We believe that a compressibility-based approach could account for a connection between these processes. However, this would involve a memory test different than the one used in the present study, such as the single-item-memory-probe test (instead of serial report) to make the different procedures match (Ricker & Cowan, 2014).

## Data Availability

https://doi.org/10.5281/zenodo.1216986

## Footnotes

Not all chunking processes are akin to a compression process: pair-associated word learning can for instance be used to chunk two words with no reduction of information in long-term memory. The only virtual reduction of information would be assumed by the use of a ‘pointer’ (in immediate memory) to the long-term memory representation. Conversely, not all compression processes are as simple as a chunking process since many options exist to recode information.

The .25 value derives from the number of trials per length, which is 4 in our case. This method corresponds to the all-or-nothing method described in Conway et al. (2005), which is a good compromise between (1) computing a span simply based on the longest sequence attained by the participant and (2) computing a score by partially crediting the correct items reported within a trial that was failed (for instance due to omission, permutation, and intrusion errors within a trial; see Mathy & Varré, 2013)

JASP. (2014). Retrieved from http://jasp-stats.org/.
